# Evaluating the Non-conventional Achalasia Treatment Modalities

**DOI:** 10.3389/fmed.2022.941464

**Published:** 2022-06-24

**Authors:** Francisco Tustumi

**Affiliations:** ^1^Department of Gastroenterology, Universidade de São Paulo, São Paulo, Brazil; ^2^Department of Surgery, Hospital Israelita Albert Einstein, São Paulo, Brazil

**Keywords:** achalasia, esophageal diseases, esophageal motility disorders, esophagus, therapeutics

## Abstract

**Introduction:**

Achalasia is a primary esophageal dysmotility disorder. Despite the high volume of studies addressing the conventional treatments for achalasia, few are debated regarding the non-conventional treatments, such as cardiectomy, cardioplasty, endoluminal substances injection (ethanolamine oleate, polidocanol, botulinum toxin), stents, and certain drugs (beta-agonists, anticholinergic, nitrates, calcium channel blockers, and phosphodiesterase inhibitors).

**Methods:**

A critical review was performed.

**Results:**

Endoscopic, surgical, and pharmacological treatments were included. A qualitative synthesis was presented.

**Conclusion:**

Non-conventional therapeutic options for treating achalasia encompass medical, endoscopic, and surgical procedures. Clinicians and patients need to know all the tools for the management of achalasia. However, several currently available studies of non-conventional treatments lack high-quality evidence, and future randomized trials are still needed.

## Introduction

Achalasia is a primary esophageal dysmotility disorder (1). This disease is incurable, and the main aim of the treatment is to provide symptoms palliation (2) or to treat the complications related to achalasia, such as cancer (3).

There is rich literature on conventional treatments for achalasia, comprising pneumatic dilation, peroral endoscopic myotomy (POEM), and laparoscopic or robotic-assisted cardiomyotomy (4–7). In the 2018 ISDE guideline (4), Heller myotomy with a partial fundoplication and POEM were considered equally effective in controlling symptoms, including dysphagia, and were considered first-line therapy for most Chicago type I and type II achalasia. Patients submitted to POEM should be advised of the gastroesophageal reflux risk. The pneumatic dilatations were considered effective, but patients desiring long-term symptoms remission may be more appropriately referred to surgery or POEM. Sigmoid shaped megaesophagus should not be indicated for endoscopic therapy (4, 8).

Despite the high volume of papers addressing the conventional treatments for achalasia, few are debated regarding the non-conventional treatments. Clinicians should know all the available tools for achalasia management, and understanding all the therapeutic possibilities is essential to better share the decisions with the patients. Consequently, this study aims to review the literature on unconventional treatments for achalasia to present the current evidence and qualify their uses. These include medical, surgical, and endoscopic non-conventional modalities for achalasia management.

## Methods

A literature review was carried out, gathering the non-conventional treatments of achalasia. The following search terms were used: “achalasia”, “treatment”, “management”, “therapeutic”, “procedure”, “surgery”, “endoscopy”, “pharmacology”, “therapy”, “esophagectomy”, “oesophagectomy”, “esophageal resection”, “cardioplasty”, “esophagocardioplasty”, “cardiectomy”, “esophagocardiectomy”, “anticholinergic”, “serotonin”, “calcium channel blockers”, “nitrates”, “phosphodiesterase”, “benzodiazepines”, “ethanolamine oleate”, “polidocanol”, “botulinum”, and “stent”, in order to overview all treatments used in achalasia. PubMed, Embase, Lilacs/BVS, Cochrane Central, and Google Scholar were the main databases searched. The research design included any observational or experimental study in humans and animal models. We considered a non-conventional treatment for achalasia any strategy other than the most applied therapeutic methods POEM, cardia dilation, and cardiomyotomy.

The following information was extracted: type of treatment (surgical, endoscopic, or medical), outcomes of the included studies (short- and long-term efficacy, and adverse events related to treatment).

The outcomes were critically evaluated with the grade of recommendation (Table 1) and Oxford level of evidence for therapeutic interventions (9) (Table 2).

**Table 1:** GRADE of recommendation (9).

**Grade**	**What it means**
A	Consistent level 1 studies
B	Consistent level 2 or 3 studies or extrapolations from level 1 studies
C	Level 4 studies or extrapolations from level 2 or 3 studies
D	Level 5 evidence or troublingly inconsistent or inconclusive studies of any level

**Table 2:** Oxford level of evidence for therapeutic studies (9).

**Level**	**What it means**
1a:	Systematic reviews (with homogeneity) of randomized controlled trials
1b:	Individual randomized controlled trials (with narrow confidence interval)
1c:	All or none randomized controlled trials
2a:	Systematic reviews (with homogeneity) of cohort studies
2b:	Individual cohort study or low quality randomized controlled trials (e.g. <80% follow-up)
2c:	“Outcomes” Research; ecological studies
3a:	Systematic review (with homogeneity) of case-control studies
3b:	Individual case-control study
4	Case-series (and poor quality cohort and case-control studies)
5	Expert opinion without explicit critical appraisal, or based on physiology, bench research or “first principles”

## Results

The present review compiled the non-conventional treatments for achalasia and qualified their effectiveness and safety according to the currently available evidence. Endoscopic, surgical, and pharmacological treatments were included. A total of 80 articles were used in this review. The Table 3 summarizes the main non-conventional therapeutic options for achalasia.

**Table 3:** Summary of the main non-conventional therapy for achalasia.

**Type**	**Non-conventional treatment for achalasia**	**Mechanism of action**	**Examples**	**Main adverse events**	Possible indications*	**Main alternatives**
Surgical	Cardiectomy	GEJ resection to facilitate esophageal emptying	Merendino procedure	Leakage	End-stage achalasia after failure of conventional therapy	Esophagectomy
	Cardioplasty	GEJ surgical modification to facilitate esophageal emptying	Thal and Serra-Dória procedures	Leakage	End-stage achalasia after failure of conventional therapy	Esophagectomy
Endoscopic	Sclerosing agents	LES sclerosis and excitatory neuron injury	Ethanolamine oleate and polidocanol	Esophageal stenosis	Patients unfit for surgery	Cardia endoscopic dilation
	Neurotoxin	Blockage of neurons acetylcholine release at the neuromuscular junction in the LES, reducing LES pressure	Botulinum toxin	Gastroesophageal reflux	Bridge therapy for patients unfit for surgery	Cardia endoscopic dilation
	Stent	Keep LES open	Self-expanding metal and biodegradable stents	Stent migration	Patients unfit for surgery	Cardia endoscopic dilation
Medical	Beta-agonists	Smooth muscle relaxants, inducing pressure reduction in the LES	Carbuterol	Dizziness, nausea	Patients with dysphagia not desiring for invasive procedures	POEM, cardiomyotomy, cardia dilation
	Anticholinergic	Smooth muscle relaxants, inducing pressure reduction in the LES	Cimetropium bromide	Dryness in mouth, constipation	Patients with dysphagia not desiring for invasive procedures	POEM, cardiomyotomy, cardia dilation
	Phosphodiesterase inhibitors	Smooth muscle relaxants, inducing pressure reduction in the LES	Sildenafil	Flushing, headache	Patients with dysphagia not desiring for invasive procedures	POEM, cardiomyotomy, cardia dilation
	Nitrates	Smooth muscle relaxants, inducing pressure reduction in the LES	Isosorbide dinitrate	Headache, palpitation, and fainting	Patients with dysphagia not desiring for invasive procedures	POEM, cardiomyotomy, cardia dilation
	Calcium channel blockers	Smooth muscle relaxants, inducing pressure reduction in the LES	Nifedipine	Headache	Patients with dysphagia not desiring for invasive procedures	POEM, cardiomyotomy, cardia dilation

*Non-conventional achalasia treatment modalities should only be considered in specific and individual situations. GEJ, gastroesphageal junction; LES, Lower esophageal sphincter.

### Surgical Options

Only a small bulk of evidence addressing alternative surgical procedures for achalasia is found in the literature. The evidence is limited to case series or small sample size cohorts. Randomized controlled trials are absent.

#### Cardiectomy

Cardia resection (cardiectomy) may facilitate esophageal emptying. Ithurralde et al. (10) analyzed the course of five achalasia patients submitted to gastroesophageal junction resection and Roux-en-Y reconstruction after failed cardiomyotomy. All the patients reported dysphagia amelioration at a mean follow-up of 34 months (Level 4; Grade: C).

Some authors proposed a surgical technique to replace the non-functional lower esophagus with a “neosphincter”. The idea was to enable esophageal emptying and avoid reflux. Merendino and Dillard (11) proposed a cardiectomy plus jejunal interposition between the esophagus and stomach.

A recently published cohort compared 22 patients who submitted to the Merendino procedure and 17 patients who submitted to a gastric conduit. The gastric conduit group had a significantly longer length of hospital stay (35.9 *vs*. 18.2 days) and a higher rate of anastomotic leakage (24% *vs*. 9%) (Level 2a; Grade: B) (12). However, there are major concerns with this cohort. The sample was small and included not only achalasia, and with high interpatient heterogeneity. There is also great concern regarding the external validity of the findings of this study. Consequently, it is reasonable to recommend that the Merendino procedure should be performed only in the setting of research protocols, with ethical approval and patients' signature of informed consent (Level 5; Grade: D).

#### Cardioplasty

Some authors proposed a cardioplasty instead of a cardiectomy. Several techniques have been reported, such as the Serra-Dória and Thal procedure. The idea was to modify the lower esophageal sphincter to facilitate esophageal emptying.

Thal et al. (13) described a type of cardioplasty that creates an anti-reflux mechanism. Thal procedure was initially described for reconstruction in esophageal distal rupture or stenosis but was later used in achalasia. Thal procedure consists of opening all the cardia layers of the wall. Then, the cardia opening is closed with a gastric fundus superposition.

Alves et al. (14) described their experience with a modified Thal procedure for achalasia. Of the 29 patients, 86% presented a resolution of all symptoms, but half of the patients showed pathological reflux at the pHmetry evaluation. There was no early postoperative mortality, but some patients died of esophagogastric cancer during long-term follow-up (Level 4; Grade: C).

Senra et al. (15) reported their experience with laparoscopic cardioplasty. The length of hospital stay was lower than 2 days, and no early complication was found. However, at long-term follow-up, all patients presented gastroesophageal reflux (Level 4; Grade: C). Griffiths et al. (16) also presented their laparoscopic cardioplasty case series. All the three investigated patients showed symptoms relief and esophageal emptying, but 2 demanded anti-reflux medication (Level 4; Grade: C). Dehn et al. (17) also pointed to gastroesophageal reflux as a long-term concern after laparoscopic stapled cardioplasty (Level 4; Grade: C).

Serra Dória et al. (18), in order to reduce the gastroesophageal reflux in patients operated on for megaesophagus, adopted a new surgical approach. They associated a cardioplasty with subtotal gastrectomy with Roux-en-Y.

Costa et al. (19) reported results of 8 patients treated with Serra-Dória after cardiomyotomy failure. All patients presented satisfactory symptom relief. In Costa et al.'s study, Serra-Dória had similar symptom control to redo cardiomyotomy (Level 4; Grade: C). However, a type-II error is likely due to the small sample size.

Aquino et al. (20) showed a 26.3% (out of 19) complication rate after the Serra-Dória procedure, including pneumonia and anastomotic leak (Level 4; Grade: C).

Braghetto et al. (21) reported a 25% leakage rate but no mortality. Dysphagia improved in 11 out of 12 patients, all of whom gained weight.

Cardioplasty theoretically could be performed as an alternative for esophagectomy or as a rescue operation during attempted cardiomyotomy following multiple perforations of mucosa (Level 5; Grade: D). Roux-en-Y could be considered to avoid severe esophagitis.

#### Esophagectomy

Esophagectomy for end-stage achalasia is the most studied surgical procedure following cardiomyotomy. Some authors may not classify esophagectomy as a non-conventional method for achalasia. However, for this manuscript, we considered a non-conventional treatment for achalasia any strategy other than the most used therapeutic methods (POEM, cardia dilation, and cardiomyotomy). Esophagectomy is not the first-choice therapy for most achalasia patients, and it is a choice exception strategy. The 2018 ISDE guideline (4) recommends esophagectomy for patients with persistent or recurrent achalasia after the failure of previous less invasive treatments and radiologic progression of the disease.

Most of the current knowledge and surgeons' experience on esophagectomy comes from cancer treatment. Esophagectomy for achalasia and cancer have both similar postoperative outcomes, including the morbidity and the rate of reoperations (Level 2c; Grade: B) (22).

Aiolfi et al. (23) performed a meta-analysis assessing the postoperative outcomes after esophagectomy for achalasia. Among the included studies, esophagectomy was performed through a transthoracic (79%) or a transhiatal (21%) approach. The stomach was the favored substitute for reconstruction (95%). The main complications reported were pneumonia (10%) and anastomotic leak (7%). The mortality rate was 2% (Level 3a; Grade: B).

Transhiatal esophagectomy may be performed by laparoscopy or open access (Level 1b; Grade: A) (24). Mediastinoscopy may help minimally invasive transhiatal esophagectomy (Level 4; Grade: C) (25).

Tassi et al. (26) compared 32 patients submitted to Heller-Dor with a pull-through technique with 16 patients submitted to esophagectomy after failed cardiomyotomy in a long-term follow-up. No differences were noted for reflux and esophagitis. Quality of life was poorer in the esophagectomy group for the domains of physical, role emotional, vitality, social functioning, and mental health. The authors advocate that cardiomyotomy should be the first-choice therapy for end-stage achalasia (Level 2b; Grade: B).

Esophagectomy, cardiectomy, or cardioplasty should be considered only in end-stage megaesophagus with recurrent dysphagia after conventional therapy. Besides, these procedures should be performed only in high-volume institutions by high experienced upper gastrointestinal surgeons (Level 2b; Grade: B) (26, 27). Even for sigmoid-shaped achalasia, the Heller-myotomy with a pull-through technique should be preferred over esophagectomy whenever it is possible (Level 2a; Grade: B) (8).

The great advantage of esophagectomy over the other surgical modalities is that esophagectomy avoids the risk of malignization (3). Consequently, esophagectomy could also be considered in high cancer risk achalasia patients (Level 5; Grade: D). The surgeon and institutional experience should be taken into account, mainly due to the lack of robust evidence for surgical procedures for achalasia other than cardiomyotomy.

### Endoscopic Options

#### Ethanolamine Oleate

Ethanolamine oleate (EO) is a substance resulting from the synthetic mixture of ethanolamine and oleic acid. EO acts as a sclerosing agent that produces local inflammatory response and, subsequently, tissue fibrosis (28). This sclerosing agent is generally used to treat vascular lesions and varices (28). It is assumed that the EO injection in the lower esophageal sphincter may induce excitatory neuron injury, provoking a predominance of inhibitory activity and reduced sphincter pressure (29).

Five original studies of the use of EO for achalasia were found. Moreto et al. (29), in a non-controlled trial, first reported the use of EO for achalasia. Third-three patients were treated with injection of EO at the cardia. Moreto et al. concluded that symptom relief was “good” or “excellent” for almost all patients, although some patients needed repeated EO injections to reach success. The symptom relief persisted for months to years. However, 20% of the patients developed some level of stricture that demanded balloon dilation (Level 2b; Grade: B). The same authors repeated the experiments in a more recent paper, showing that the cumulative expectancy of being free of recurrence was 90% at 50 months with EO (30) (Level 2b; Grade: B).

Niknam et al. (31, 32) applied EO to patients that were poor candidates for cardia dilation or cardiomyotomy. The authors also concluded that EO provides good symptom control, but some patients may demand reinjection (Level 4; Grade: C). The main adverse events were chest pain and erosion in the distal esophagus.

Mikaeli et al. (33) presented a prospective controlled study, including patients unfit for surgery or dilation. The authors concluded that EO has comparable efficacy to botulinum toxin injection for the treatment of achalasia (Level 1b; Grade: A).

Consequently, EO injection in the lower esophageal sphincter may be an option for patients unfit for surgery as an alternative option for cardia dilation. Repeated injection may be needed, and patients should be aware of the risk of stricture and local erosion. The routine use of EO is not advised due to the low number of published papers.

#### Polidocanol

As well as EO, polidocanol is a sclerosing agent, and its endoscopic injection in the lower esophageal sphincter has been proposed to treat achalasia (34).

Two studies evaluate the use of polidocanol in achalasia.

When compared to EO, polidocanol seems to be less effective. Although both sclerosing agents show dysphagia relief reduction in the esophageal sphincter pressure, the long-term treatment failure of polidocanol is higher than that of EO (Level 2b; Grade: B) (30).

However, in the short- and middle-term (6 months), polidocanol seems to be more efficient than botulinum toxin injection, with better symptom control and less need for rescue therapy (surgery or dilation) (Level 1b; Grade: A). (35).

As well as EO, polidocanol injection in the lower esophageal sphincter could be an option for patients unfit for surgery as an alternative for cardia dilation. However, results may be worse than EO in long-term follow-up. The routine use of polidocanol is not advised due to the low number of published papers.

#### Botulinum Toxin Injection

The botulinum toxin A (BTX) is a neurotoxin that induces blockage of neurons acetylcholine release at the neuromuscular junction, promoting muscle paralysis (36). BTX has been used in the lower esophageal sphincter, promoting the reduction of sphincter contraction, and facilitating esophageal food transit. 100 units of BTX above gastroesophageal junction are enough to produce the desired effect and can be performed as a day case procedure. (37).

Indeed, among the substances that may be injected into the lower esophageal sphincter to reduce its pressure, the BTX is the most studied. Consequently, the efficacy and complications related to this procedure are better known. Severe complications were reported for BTX (Level 4; Grade: C). By inference, we can estimate that some of these complications can also be seen for EO and policanol injection (Grade: D). The severe complications related to BTX included hepatic (38) or subphrenic abscess (39), esophageal perforation, mediastinitis, and thoracic aorta pseudoaneurysm (40–42) (Level 4; Grade: C). The use of echo-guided injection can be considered to avoid severe complications, although there is no clear evidence of the superiority of the echo-guided over blinded injection regarding safety (Level 2b; Grade: B) (43).

The BTX application in the lower esophageal sphincter diffuses into the hiatus and causes its paresis (Level 2b; Grade: B) (44). This paralysis may induce severe gastroesophageal reflux and esophagitis (Level 2b; Grade: B) (44, 45). Other common adverse events include chest pain and heartburn (Level 2b; Grade: B) (40).

BTX has a short-duration efficacy. In a case series, Yamaguchi et al. (46) reported high dysphagia relief by 1-week therapy, but 50% of the patients relapsed at 3–24 months after treatment (Level 4; Grade: C). Due to the short-duration efficacy, authors usually propose BTX use only for achalasia patients unsuitable for more definitive procedure as a bridge therapy (Level 2b; Grade: B) (47–51). Some case reports suggested the use of BTX for achalasia during pregnancy (Level 4; Grade: C) (52–54). Some authors also suggest endoscopic ultrasound-guided BTX injection for treating achalasia patients with esophageal varices (Level 4; Grade: C) (50, 55). Theoretically, due to the short-term efficacy, BTX could also be used as a therapeutic test for uncertain esophageal dysmotility conditions (Level 5; Grade: D).

In a recent network meta-analysis (56), BTX injection was considered far the treatment option with the lower efficacy if compared with cardiomyotomy, POEM, pneumatic dilation, and mixed methods (Level 1a; Grade: A). BTX has poorer outcomes even when stratified according to each achalasia subtype (Level 2a; Grade: B) (57).

Comparing the efficacy of the BTX and pneumatic dilation, no difference is found at 1-month follow-up. However, after 6 months, the relapse rate in the BTX group is higher than in the dilation group (Level 1a; Grade: A) (58). Besides, there is no difference between BTX and pneumatic dilation regarding safety (Level 2b; Grade: B), (59), and consequently, pneumatic dilation should be preferred over BTX injection (Level 2b; Grade: B).

Zagory et al. (60) compared BTX and Heller myotomy for children with achalasia. The authors found superiority of the Heller myotomy group for controlling symptoms as first-line therapy for achalasia (Level 2b; Grade: B).

Mikaeli et al. (33) found, in a prospective controlled study, that BTX injection and EO injection have comparable efficacy (Level 1b; Grade: A). However, in long-term follow-up, sclerotherapy modalities are more efficient than botulinum toxin injection (Level 1b; Grade: A). (35).

Cai et al. (61) compared BTX endoscopic injection and removable self-expanding metal stents for achalasia (SEMS). The authors concluded that SEMS has a higher efficacy for controlling symptoms at 12 and 36 months of follow-up. However, SEMS were associated with adverse events such as chest pain, regurgitation, and stent migration (Level 2b; Grade: B).

The 2018 ISDE guideline (4) recommends BTX only as a bridge to a more effective therapy. The BTX injection is a choice exception strategy, and should reserved for patients unfit for surgery, POEM, or endoscopic dilation.

#### Esophageal Stent

The esophageal stent has been used for esophagus obstruction, mainly for malignant conditions (62). Stents for achalasia have been poorly studied in the past years.

Comparing pneumatic dilation and esophageal stenting for achalasia, the current evidence is conflicting. Qian et al. (63) in a retrospective cohort compared pneumatic dilation (*n* = 76) *vs*. stenting (*n* = 75). The authors concluded that both modalities have similar efficacy at short-term follow-up, but stenting shows better symptom relief after 1 year of follow-up (Level 2b; Grade: B). In another cohort, Zhao et al. (64) compared 41 patients who submitted to balloon dilation and 47 that underwent metal stent placement for achalasia. No difference between groups was found, despite a slight non-statistically significant tendency favoring stenting long-term efficacy (Level 2b; Grade: B). Dai et al. (65) proposed using a modified form of retrievable, self-expandable, nickel-titanium alloy stent. Patients with the modified stent showed better symptom control at 6 months than balloon dilation (Level 2b; Grade: B).

Cai et al. (61) compared BTX endoscopic injection and removable self-expanding metal stents for achalasia (SEMS). The authors concluded that SEMS has a higher efficacy for controlling symptoms at 12 and 36 months of follow-up (Level 2b; Grade: B). However, SEMS were associated with adverse events such as chest pain, regurgitation, and stent migration (Level 2b; Grade: B) (61, 66). Some authors suggest endoscopic suture fixation of esophageal stents to avoid migration (Level 2b; Grade: B) (67).

Hernandez-Mondragon et al. (68) proposed the use of biodegradable stents in octogenarian patients with achalasia in a non-controlled clinical trial. Biodegradable stents are made of a resorbable polymer. The authors concluded that biodegradable stents have a 65.4% clinical success rate in an intention-to-treat analysis (Level 2b; Grade: B).

A sigmoid-shaped megaesophagus should not be indicated for any endoscopic therapy since the esophageal axis can not be properly corrected with endoscopy (4, 8). The 2018 ISDE guideline recommends against temporary (absorbable or retrievable) stents and intersphincteric injection with sclerotherapy for achalasia due to the low volume of scientific papers (4).

### Medical Options

Medical treatment for achalasia has also been proposed. Candidate drugs act as smooth muscle relaxants, inducing pressure reduction in the lower esophageal sphincter.

#### Beta-Agonists

Beta-agonists simulate the functions of the catecholamines and promote bronchodilation. They are typically used for asthma and chronic obstructive pulmonary disease (69). Beta-agonists decrease the esophageal sphincter retention pressure (Level 2b; Grade: B) (70). This phenomenon is usually seen as an adverse event for patients treating respiratory conditions, such as chronic obstructive pulmonary disease, favoring esophageal reflux episodes (71). One old study evaluated beta-agonists in achalasia patients and showed a reduction in esophageal sphincter pressure lasting over 90 min (Level 2b; Grade: B) (72).

#### Serotonin and Norepinephrine Reuptake Inhibitors

Serotonin and norepinephrine reuptake inhibitors are antidepressants and act by binding to the serotonin and norepinephrine transporters (73). Serotonin and norepinephrine reuptake inhibitors also act in the lower esophageal function (Level 2b; Grade: B) (74, 75). However, no study addresses their use in achalasia patients, and consequently, their use should be restricted to research protocols (Level 5; Grade: D).

#### Benzodiazepines and Opioids

Opioids inhibit excitatory neurotransmitter release (76), and benzodiazepines inhibit smooth muscle contraction (77). Benzodiazepines and opioids are associated with elevated integrated relaxation pressure (Level 1b; Grade: A) (75, 78). However, no study addresses their use in achalasia patients, and their administration should be considered only for research protocols (Level 5; Grade: D).

#### Anticholinergic

Anticholinergic drugs are used for the management of numerous diseases, such as Parkinson's disease, urinary incontinence, cardiorespiratory conditions, and others. Anticholinergic medications block the action of acetylcholine (79). In the esophagus, they act by improving peristalsis and reducing sphincter pressure. Marzio et al. (80), in an old controlled trial, reported the efficacy of cimetropium bromide in achalasia, and the effect was maintained for 45 min (Level 1b; Grade: A).

#### Phosphodiesterase Inhibitors

Phosphodiesterase inhibitors are commonly used for erectile dysfunction, heart failure, and airway conditions (81). Phosphodiesterase inhibitors hydrolyze cyclic nucleotides and regulate cell function through cAMP and cGMP pathways. The most common drugs in this category are theophylline, zaprinast, sildenafil, tadalafil, and vardenafil.

Sildenafil lowers sphincter pressure and propulsive forces in the body of the esophagus of healthy subjects (82). Bortolotti et al. (83), in a small sample size randomized trial of idiopathic achalasia patients, showed that a 50-mg tablet of sildenafil lowered esophageal sphincter tone and the effect lasted <1 h. (Level 1b; Grade: A).

#### Nitrates

Nitrates act by releasing nitric oxide, which activates the enzyme guanylate cyclase, leading to smooth muscle relaxation. They include isosorbide dinitrate, nitroglycerin, amyl nitrate, and octyl nitrate and are usually applied in cardiovascular medical conditions (84).

Isosorbide dinitrates are taken sublingually (2.5–5mg). Isosorbide lowers the esophageal sphincter pressure and promotes esophageal emptying in the megaesophagus (Level 2a; Grade: B) (85). Side effects are common and include headache, palpitation, and fainting. The incidence of headache is over 30% (Level 2a; Grade: B) (85).

#### Calcium Channel Blockers

The calcium channel blockers inhibit inward calcium flux through ion-specific channels in the cell wall. With low intracellular calcium, smooth muscle cells relax. Calcium channel blockers comprise nimodipine, nilvadipine, nitrendipine, isradipine, and nifedipine (86).

In the megaesophagus, nifedipine (10–20 mg, sublingually) promotes the reduction of sphincter pressure but does not change esophageal emptying. Headache is a common side effect (10.5%) (Level 2a; Grade: B) (85).

A few published articles address medical treatment for achalasia, making this therapeutic option of concern. Drugs acting in the esophagus are usually seen only as adverse events for other disease treatments and not as therapeutic choices for achalasia. Theoretically, patients with mild dysphagia due to achalasia not desiring to be submitted to invasive procedures could benefit from medications before meals. However, oral intake drugs may not be absorbable in esophageal stasis, and erratic absorption could prone achalasia patients to a significant risk for complications (Level 5, Grade: D). Oral medical therapy should not be routinely offered to advanced megaesophagus, mainly those patients with significant stasis (Level 5, Grade: D).

## Discussion

The present study reviewed the main non-conventional therapeutic options for treating achalasia. Most of the included original studies presented a low level of evidence and low certainty assessment. Consequently, conventional achalasia treatments, that are supported by a higher number of evidence, including esophageal dilation, cardiomyotomy, and POEM, should be preferably chosen as the first-line treatment. However, clinicians and patients should be aware of the non-conventional treatment options. Patients should be aware of the possibilities and limitations of each treatment option.

The present review draws attention to the need for future studies in esophageal treatment. Several treatment options were poorly studied, and future controlled trials should bring a higher level of evidence to support any decision-making.

This study has some limitations. Although the review process comprises steps typically found in a systematic review, such as multiple databases searching and critical appraisal, this is not a pure systematic review. We decided on a more malleable structure review, allowing a broader thematic approach, giving the possibility to combine different methods and design studies with critical view. Systematic reviews hinder the synthesis of findings of different types of studies. The current review includes different research types, but all focused on the same topic to generate evidence to guide decision-making. However, non-systematic reviews are prone to a higher risk of selection bias. Future studies are still needed, and only after high-quality original studies with focused, therapeutic interventions a well-performed systematic review will be possible.

## Conclusion

Non-conventional therapeutic options for treating achalasia encompass medical, endoscopic, and surgical procedures. Clinicians and patients need to know all the tools for achalasia management. However, several currently available studies of non-conventional treatments lack high-quality evidence, and future randomized trials are still needed. Based on current literature, non-conventional achalasia treatment modalities should only be used in specific and individual situations.

## Author Contributions

FT: searching, extracting, writing, and reviewing. The author confirms being the sole contributor of this work and has approved it for publication.

## Conflict of Interest

The author declares that the research was conducted in the absence of any commercial or financial relationships that could be construed as a potential conflict of interest.

## Publisher's Note

All claims expressed in this article are solely those of the authors and do not necessarily represent those of their affiliated organizations, or those of the publisher, the editors and the reviewers. Any product that may be evaluated in this article, or claim that may be made by its manufacturer, is not guaranteed or endorsed by the publisher.
